# iCanCope PostOp: User-Centered Design of a Smartphone-Based App for Self-Management of Postoperative Pain in Children and Adolescents

**DOI:** 10.2196/12028

**Published:** 2019-04-22

**Authors:** Kathryn A Birnie, Fiona Campbell, Cynthia Nguyen, Chitra Lalloo, Argerie Tsimicalis, Clyde Matava, Joseph Cafazzo, Jennifer Stinson

**Affiliations:** 1 Child Health Evaluative Sciences The Hospital for Sick Children Toronto, ON Canada; 2 Lawrence S Bloomberg Faculty of Nursing University of Toronto Toronto, ON Canada; 3 Department of Anesthesia and Pain Medicine The Hospital for Sick Children, University of Toronto Toronto, ON Canada; 4 Ingram School of Nursing Faculty of Medicine McGill University Montreal, QC Canada; 5 Shriners Hospitals for Children - Canada Montreal, QC Canada; 6 Centre for Global eHealth Innovations Techna Institute University Health Network Toronto, ON Canada; 7 Institute of Health Policy, Management, and Evaluation Dalla Lana School of Public Health University of Toronto Toronto, ON Canada; 8 Institute of Biomaterials and Biomedical Engineering University of Toronto Toronto, ON Canada

**Keywords:** postoperative pain, smartphone, mobile applications, mHealth, pain management, self-management, adolescent

## Abstract

**Background:**

Moderate to severe postoperative pain in children is common. Increased pediatric day surgeries have shifted postoperative pain management predominantly to the home setting. Mobile health technology has the potential to overcome barriers to pain care by improving access to self-management resources. However, pain apps generally lack scientific evidence and are highly underutilized due to lack of involvement of end users in their development. Thus, an evidence-based pain self-management smartphone app that incorporates the needs and perspective of children and adolescents (end users) has potential to improve postoperative pain management.

**Objective:**

This paper aimed to describe how the principles of user-centered design were applied to the development of *iCanCope PostOp*, a smartphone-based pain self-management app for children and adolescents after surgery. Specifically, it presents 2 completed phases of the user-centered design process (concept generation and ideation) for the *iCanCope PostOp* app.

**Methods:**

Phase 1 was a multisite needs assessment from the perspective of 19 children and adolescents who had undergone various day surgeries, 19 parents, and 32 multidisciplinary health care providers. Children, adolescents, and parents completed individual semistructured interviews, and health care providers participated in focus groups. Data were summarized using qualitative content analysis. Phase 2 developed a pain care algorithm for the app using Delphi surveys and a 2-day in-person design workshop with 11 multidisciplinary pediatric postoperative pain experts and 2 people with lived experience with postoperative pain.

**Results:**

Phase 1 identified self-management challenges to postoperative pain management and recovery; limited available resources and reliance on medications as a predominant postoperative pain management strategy; and shared responsibility of postoperative pain care by children and adolescents, parents, and health care providers. Key app functions of tracking pain, pain self-management strategies, and goal setting were identified as priorities. Phase 2 led to the successful and efficient generation of a complete preliminary pain care algorithm for the *iCanCope PostOp* app, including clinically relevant inputs for feasible assessment and reassessment of pain and function (rest or sleep, movement or play, and mood or worry), as well as a catalog of pain management advice to be pushed to end users (psychological, physical, pharmacological, and education).

**Conclusions:**

The concept ideation and generation phases of the user-centered design approach were successfully completed for the *iCanCope PostOp* app. Next steps will include design finalization, app development (iOS or Android), evaluation through a randomized controlled trial, and subsequent implementation of the *iCanCope PostOp* app in clinical care.

## Introduction

### Background

More than 3 million children and adolescents in Canada and the United States undergo surgery each year [1-3]. Despite the availability of evidence-based clinical practice guidelines for postoperative pain [4], children and adolescents continue to experience moderate to severe pain once home in the days following outpatient surgery [5]. Unrelieved or undertreated postoperative pain can delay remobilization, lead to increased opioid use, and negatively impact health-related quality of life, including sleep, anxiety, social, and school functioning [6-10]. Furthermore, approximately 20% of children undergoing surgery develop chronic pain [11], an expensive and debilitating health problem [12].

Due to health system changes, an increasing number of pediatric surgical procedures are performed as day surgeries [1], resulting in greater postoperative pain management within the home setting. Problematic, however, are reports that parents infrequently give postoperative pain medications to their children after hospital discharge [13]. Moreover, children and adolescents feel challenged by the need to rely on others, such as nurses, for assistance with postoperative pain relief [14]. Greater confidence in one’s ability to manage pain (“pain coping self-efficacy”) contributes to decreased postoperative pain and lowered risk for chronic pain in children [6,11], and is a mechanism by which effective disease self-management is achieved [15].

Growing evidence supports the use of mobile health (mHealth) technology to improve disease self-management [16]. Smartphones are widely used by children and adolescents, with 88% having access to a mobile device [17]. Despite this availability, relatively few apps have been designed for children and adolescents to support disease self-management [18]. mHealth apps have shown potential for improving postoperative symptom monitoring and self-management in adults [19]; however, none currently exist for children and adolescents [20]. Estimates from a 2017 report of digital health indicated that over 318,000 mHealth apps are available worldwide with more than 200 new health apps added daily [21]; however, more than 85% of apps have less than 5000 downloads, suggesting that users do not find them relevant or effective [22]. Also notable is the lack of scientific evidence underlying mHealth apps, particularly among apps for pain [21,23]. A recent scoping review of all apps for postoperative pain revealed that none had comprehensive pain or self-management content, had undergone scientific evaluation, or had involved end users in their development [20].

### User-Centered Design Process for Mobile Health App Development

User-centered design is a collaborative evidence-based approach that incorporates the needs and context of a specific end-user group to inform the development and design of mHealth apps [24,25]. General steps in user-centered design include understanding the environment in which the app will be used and end-user needs; evaluating and applying relevant theory, design science, and content area evidence; as well as iteratively producing and evaluating app prototypes and the final product [24,25]. Typically, an iterative process with multiple diverse user-centered design qualitative research methods is used for the design and development of the app, including focus groups, interviews, participatory design sessions, and usability testing, with randomized controlled trials to evaluate app efficacy [24,25]. Consideration of the end-user experience and their involvement at every stage of the design process closes the research-practice gap by producing apps that are patient-centered, effective, novel, feasible, and acceptable in daily life [22,24,25]. The focus on end-user concerns ensures individual relevance and tailoring for effective disease self-management [15]. Thus, application of user-centered design to the development of a self-management app has the potential to critically improve postoperative pain care for children and adolescents at home.

**Figure 1:**
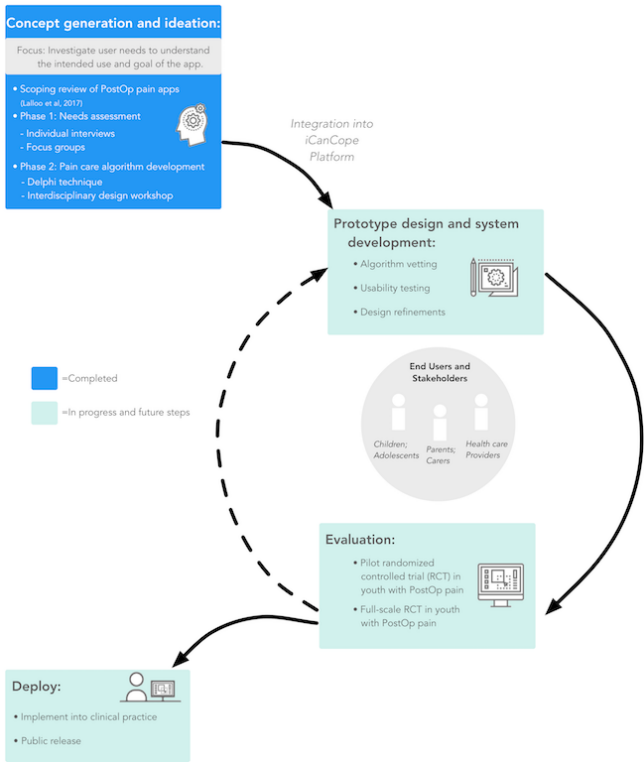
Steps in the user-centered design process. PostOp: postoperative; RCT: randomized controlled trial.

This paper presents an overview of the user-centered design process in developing “iCanCope with Postoperative Pain” (*iCanCope PostOp*), a smartphone-based app for children and adolescents’ self-management of acute postoperative pain (see Figure 1). This paper focuses on results from 2 completed early design phases that fall within the user-centered design: the first stage of concept generation and ideation (phase 1) involves a needs assessment from the perspective of children and adolescents who have undergone any type of day surgery (app end users), parents, and health care providers; and the second phase involves (phase 2) development of a preliminary evidence-based expert-generated pain care algorithm for the app. User-centered design and disease self-management provided evidence-based frameworks for all study methods [15,24,26]. A previously completed scoping review of postoperative pain self-management apps [20] also supports the development of *iCanCope PostOp*. Similar applications of user-centered design for pediatric pain self-management apps include those for chronic and cancer-related pain [27-29].

## Methods

### Phase 1: Needs Assessment

#### Participants

Participants included a convenience sample of 15 to 20 dyads of children and adolescents aged 8 to 18 years who had undergone surgery in the past 7 days with self-reported acute postoperative pain and a parent as well as a purposive sample of 20 to 30 health care providers recruited for variability in terms of discipline and level of experience treating acute postoperative pain. Potential patient-parent dyads were identified through a review of the operating room list and consultation with preanesthesia clinic coordinators. Eligible patients and parents were initially introduced to the study by a member of their health care team before their scheduled procedure. Patients and parents were excluded if they had severe cognitive impairments or a major medical or psychiatric illness that precluded their ability to participate in a verbal interview. Children and adolescents with an existing recurrent or chronic pain condition were also excluded, given the potential differences in managing acute postoperative pain alone versus in addition to chronic pain. Eligible health care providers had worked in the pediatric perioperative setting for at least 1 year and were not trainees. They were recruited through study invitation emails and at departmental rounds. Participants were recruited from 2 pediatric tertiary care centers, including The Hospital for Sick Children (primarily English-speaking) and Shriners Hospitals for Children, Canada (primarily French-speaking). Children, adolescents, and parents were recruited from the outpatient surgery preanesthesia clinic area. Research ethics approval was obtained from both the institutions. As parent-child dyads were recruited together, all parents provided informed consent, and children and adolescents provided informed consent or assent, as appropriate. Interviews with children, adolescents, and parents were conducted by phone. Focus groups took place at the hospitals.

#### Individual Semistructured Interviews and Focus Groups

All participants reported demographics. Individual interviews were conducted with children, adolescents, and parents, whereas focus groups were conducted with health care providers. Semistructured interview guides were informed by evidence and team expertise in mHealth [29,30], disease self-management, and pediatric postoperative pain (see Textbox 1). Interviews and focus groups were conducted in iterative cycles until data saturation was reached and explored participants’ (1) experience managing acute postoperative pain (eg, pain, challenges, resources available, and responsibility for pain management) and (2) perceptions about proposed optimal design of a smartphone-based app to support postoperative pain management, including design functions of tracking pain, pain self-management strategies, and goal setting.

**Needs assessment questions from semistructured interviews and focus groups.**

- Can you tell me about [the child’s] pain after surgery?
  - What were the biggest challenges?
- Who do you think is responsible for managing [the child’s] postoperative pain?
  - Child’s role, parents’ role, and health care providers’ role?
- Did you feel like there are adequate tools or resources needed to manage pain at home after surgery?
  - What services would you like to have available to help [the child] manage pain? For example, health care providers, education, and strategies to track or manage pain.
- Can you tell me about other troublesome symptoms that [the child] had after surgery?
  - Mood, sleep, physical activities, social activities, physical symptoms, missed school, or other symptoms?
- Did you feel like [the child] had the tools or resources that [the child] needed to manage these other symptoms at home after surgery?
- What do you think about having a smartphone app that could help better manage pain at home after surgery?
  - Would you be interested in tracking [the child’s] pain and other symptoms after surgery? Why or why not?
  - Would you want a function on the app to help [the child] set goals related to pain and function? Why or why not?
  - Would you find it useful for [the child] to have an app toolbox of ways to manage pain in the moment? Why or why not?

#### Data Analysis

Audio-recordings were transcribed verbatim. Interviews and focus groups were independently coded in NVivo (QSR International) 10 [31] by 2 research assistants fully bilingual in English and French using simple content analysis [32]. A subset of the English-language transcripts was initially reviewed by 2 research team members to establish overarching themes and develop a coding scheme informed by frameworks of effective pain and disease self-management to be iteratively revised as needed during the coding process. Interrater reliability of all coded text was calculated using percent agreement [31] as well as comparative analysis, whereby coders reviewed each other’s coded interviews followed by discussion [32]. Percent agreement from 13 (of 19) interviews and 2 (of 4) focus groups was 97%. Disagreements were resolved through consensus and consultation with a third person (study investigator), if needed.

### Phase 2: Development of the App Pain Care Algorithm

#### Delphi Survey

A 2-stage Delphi survey technique [33] was used to determine pain inputs by app end users corresponding to app features endorsed by children, adolescents, parents, and health care providers in phase 1. Clinically important pain inputs were defined as self-reported clinical characteristics by children and adolescents about their postoperative pain experience that should result in pain management advice being generated by the app. Email invitations for study participation were sent to 16 multidisciplinary researchers and health care providers identified by the research team for their recognized expertise in the assessment and management of pediatric postoperative pain. Overall, 2 survey iterations asked participants to rank the importance, or indicate the necessity of, suggested pain input items (Survey 1: 11-point scale from 0=“not at all important” to 10=“extremely important;” Survey 2: select “necessary,” “desirable,” or “not needed”) as well as possible item rewording or additional comments. Surveys were Web-based using the Vanderbuilt University REDCap survey software [34]. Invited experts were given a 2-week period to complete the surveys, with 1 email reminder. Moreover, 9 participants completed survey 1 and 14 completed survey 2. Participant demographics are reported in Table 1. Research ethics approval was obtained from The Hospital for Sick Children.

**Table 1:** Delphi survey and expert design workshop participant demographic characteristics.

Characteristics		Pain care algorithm interprofessional experts^a^ (n=13)
**Sex, n (%)**		
	Female	10 (77)
	Male	3 (33)
Age (years), mean (SD)		46.31 (13.32)
**Type of expert, n (%)**		
	Anesthesiologist	3 (23)
	Advanced practice nurse	2 (15)
	Child life specialist	1 (8)
	Surgeon	1 (8)
	Psychologist or psychology intern	2 (15)
	Physical therapist	1 (8)
	Other physician (eg, pediatrician)	1 (8)
	Person living with chronic postoperative pain^b^	2 (15)
Years of experience as a health care provider, mean (SD)		22.33 (12.23)
Years of experience in pediatrics, mean (SD)		18.00 (11.45)

^a^An additional 3 experts completed the Delphi survey, but did not attend the design workshop (psychologist, surgeon, and nurse).

^b^Not included in the Delphi survey.

#### Interprofessional Expert Design Workshop

A 2-day face-to-face interprofessional expert design workshop was held in Toronto, Canada, to develop a preliminary pain care algorithm for the *iCanCopePostOp* app including features endorsed by children, adolescents, parents, and health care providers in phase 1 as well as important pain inputs by end users, evidence-informed pain self-management advice, and end-user app flow. A total of 13 participants attended this. All participants from the Delphi survey were invited and 11 of 14 attended in addition to 2 people with lived experience with postoperative pain (1 adult and 1 adolescent). Participant demographics are reported in Table 1. Research ethics approval was obtained from The Hospital for Sick Children.

The workshop was facilitated by a member of the research team and structured based on previous successful design and consensus workshops for developing self-management apps in pediatric pain [27]. Participants were provided an overview of workshop goals, the user-centered design process (presented by 2 experts in user-centered design and development of the mHealth apps), results from previously completed app development phases (scoping review [20]; phase 1 needs assessment and phase 2 Delphi survey), and available evidence for the management of pediatric postoperative pain (eg, [35]). Small and large group discussions were used to refine clinically important pain inputs from app end users (eg, what pain inputs would trigger a pain alert to the child/adolescent?) and evidence-informed pain self-management advice (eg, what are the patient-driven pain management techniques children and adolescents should use to manage their acute postoperative pain?). mHealth experts provided input on technical practicality and feasibility of system suggestions made by workshop participants. To improve rigor and transparency of findings informing the pain care algorithm, ideas generated from the first day were summarized and presented to workshop participants for feedback on the second day. In generating the final algorithm, audio-recordings of the workshop and research team field notes were referenced.

## Results

### Phase 1: Needs Assessment

#### Participants

Demographic characteristics for children and adolescents (n=19), parents (n=19), and health care providers (n=32) are reported in Table 2. A total of 4 focus groups were conducted; 1 with 7 health care providers, 2 with 8 health care provides, and 1 with 9 health care providers. Overall, 4 patient and parent interviews and 2 of 4 focus groups were conducted in French. Children and adolescents were aged between 8 and 18 years. Health care providers were multidisciplinary and practiced across perioperative inpatient and outpatient hospital care settings, including the operating room, preanesthesia clinics, and postanesthesia care units.

**Table 2:** Needs assessment participant demographic characteristics.

Characteristics		Children and adolescents (n=19)	Parents (n=19)	Health care providers (n=32)
**Sex, n (%)**				
	Female	13 (68)	3 (16)	25 (78)
	Male	6 (32)	16 (84)	7 (22)
**Age (years)^a^, mean (SD)**		15.26 (2.51)	—^b^	—
	20 to 29, n (%)	—	3 (16)	2 (6)
	30 to 39, n (%)	—	13 (68)	5 (17)
	40 to 49, n (%)	—	3 (16)	11 (35)
	50 to 59, n (%)	—	—	8 (26)
	60 to 69, n (%)	—	—	5 (16)
**Type of day surgery, n (%)**				
	Orthopaedic	12 (63)	—	—
	Plastic	2 (10)	—	—
	Urology	5 (27)	—	—
**Ethnicity^c^, n (%)**				
	White	6 (39)	6 (39)	—
	Arabic or West Asian	2 (13)	2 (13)	—
	South Asian	2 (13)	2 (13)	—
	Black	1 (7)	1 (7)	—
	Filipino	1 (7)	1 (7)	—
	South East Asian	1 (7)	1 (7)	—
	Other	1 (7)	1 (7)	—
	Prefer not to answer	1 (7)	1 (7)	—
**Type of expert, n (%)**				
	Anesthesiologist	—	—	4 (12)
	Staff nurse	—	—	13 (41)
	Advanced practice nurse	—	—	4 (12)
	Child life specialist	—	—	3 (11)
	Surgeon	—	—	4 (12)
	Psychologist or psychology intern	—	—	0 (0)
	Physical therapist	—	—	2 (6)
	Other physician (eg, pediatrician)	—	—	0 (0)
	Occupational therapist	—	—	2 (6)
Years of experience as a health care provider, mean (SD)		—	—	20.18 (11.56)
Years of experience in pediatrics, mean (SD)		—	—	17.05 (11.75)

^a^Missing data from 1 health care provider.

^b^Not applicable.

^c^Missing data from 4 children or adolescents and 4 parents.

#### Interview and Focus Group Themes

Responses from the semistructured interviews and focus groups were categorized into 4 overarching themes: (1) challenges to managing pediatric postoperative pain, (2) available resources and postoperative pain management strategies, (3) responsibility for postoperative pain management, and (4) reactions to proposed smartphone-based app and app features to facilitate child and adolescent self-management of postoperative pain.

#### Challenges Managing Postoperative Pain

All interviewed children and adolescents reported postoperative pain, described with varying intensity from “just a little bit” to “a lot.” Children, adolescents, and parents identified more intense pain in the initial days following surgery and difficulty managing pain at home. As one parent stated:

The 24 hours after the surgery, she had severe pain...I ended up taking her to Emerg.

P-6

In addition to pain intensity, children, adolescents, and parents identified negative impacts of pain and surgical recovery on movement and mobilizing, sleep, drowsiness or fatigue, nausea, mood (feeling frustrated or bored), and worry. Parents identified additional challenges related to managing postoperative pain medications as well as uncertainty as to when to allow children to return to normal activity. Challenges identified by health care providers in managing postoperative pain included individual variability and subjectivity in pain experience (eg, intensity and response to medications). Other stated challenges were understanding each patient’s postoperative experience and context as well as effectively assessing and communicating with families about pain management once families had returned home.

#### Available Resources and Postoperative Pain Management Strategies

Children and adolescents generally identified that they had the needed resources to manage pain after surgery, although their responses reflected a reliance on medications as a sole pain management strategy. As 1 adolescent stated:

We really didn't use anything to help me, all we used was the medications.

A-2

Parents identified a desire to have greater access and contact with health care providers, if needed. Health care providers identified a need for greater ongoing resources and education to support postoperative pain management. As 1 health care provider summarized:

We are kind of pushing them out the door sometimes, without proper instructions and it’s [an] overload of information...plus they’re confused with how much pain is normal.

HCP-1

#### Responsibility for Postoperative Pain Management

Children and adolescents, parents, and health care providers all held responsibility in postoperative pain care. Some children identified themselves as primarily responsible:

...it would be myself because...I was helping my foot get better.

A-5

Other children and adolescents emphasized the primary role of health care providers and parents:

I was in the hospital and then it was the doctors and nurses...but now that I am home, [it’s] my mom.

A-7

Parents generally identified themselves in collaboration with symptom reporting from the child or adolescent:

It’s both the parents as well as the child. The child needs to tell the parent how much [pain]...so based on [what pain rating] she would tell me I would give medication.

P-2

Health care providers identified a team approach as necessary for optimal postoperative pain care. As 1 health care provider summarized:

It’s a team approach...I mean yes, everyone from the healthcare team, but the family is part of the team, and the patient is part of the team...that’s the only way that we’re going to be totally successful.

HCP-2

#### Response to Proposed Smartphone-Based App and App Features

Children, adolescents, parents, and health care providers all thought a proposed smartphone app would improve accessibility and self-management of children and adolescents in postoperative pain care.

##### Tracking Pain

All children, adolescents, and parents identified the value of tracking pain as a means of increasing communication with each other and with the health care team as well as to inform pain management strategies. As 1 adolescent stated:

It's a great idea so you don't have to remember what your pain was like 3 weeks ago after the surgery...you can have your phone and show your doctor.

A-14

Health care providers also identified symptom tracking as valuable:

Day to day activities, that’s how we measure it because depending on what [the child] can and can’t do indicates the level of the pain they’re experiencing.

HCP-9

##### Pain Self-Management Advice

All children, adolescents, parents, and health care providers saw the value of including proposed pain self-management advice within the app. Parents identified a key benefit of the app for increasing children’s and adolescents’ independence in pain management:

I think it would be great for her as a teenager to have the control and the input into how to make decisions around managing her pain.

A-13

Children and adolescents also identified the relevance of including information about surgery. For example, 1 adolescent stated:

I think it would be a good idea to explain all of the operations.

A-1

Another adolescent reported:

I could type in [the type of surgery] and search and I can just bring up all the facts about it and explain it more.

A-3

##### Goal Setting

All children, adolescents, parents, and health care providers felt inclusion of the proposed goal setting within the app would be helpful for postoperative pain and recovery, emphasizing the need for goals to be individually tailored and realistic. One adolescent shared:

It could be helpful because it would sort of be a challenge for me. I could write [the doctors’ recommendations] in and then it could be like a reminder when they pop up on the.

A-14

Goals were identified as potentially helping to address the uncertainty about return to normal function. As a health care provider summarized:

[Adolescents] usually want to know if they can return to physical activities, sports...school. Parents want to know the goal of recovery and when they can we expect less pain...[and] return to normal functioning.

HCP-10

##### Other Suggestions

Additional app features for consideration were also suggested by children, adolescents, parents, and health care providers. Specifically, the possibility of direct communication between children, adolescents, parents, and health care providers was proposed. As 1 adolescent described:

Maybe a chat system if [patients] have questions and they can easily ask the [hospital] staff if they need to.

A-12

As 1 parent stated:

What would have been really helpful for the first night and second day would probably have been if you could call a 1-800 number and get an online service...specific about post-op.

P-1

Parents also suggested including a function to track postoperative pain medications:

I think the biggest thing was some way to track the medications...when’s the next dose...because there’s so much information when your child gets surgery.

P-6

### Phase 2: Development of the App Pain Care Algorithm

#### Delphi Survey

The first survey included 21 possible pain input items with a mean item importance rating of 7.73 (SD 1.38; range mean importance of 4.88 to 9.89 per item). Rewording was suggested for all items. On the basis of participant responses and comments from the first survey iteration, 4 items were removed: least and average pain intensity, duration of pain, and impact of pain on relationships with friends and family since last entry on the app. In addition, 1 item was added to assess the impact of pain on eating and drinking. As such, a total of 18 items were included in the second survey iteration. Items and results from survey 2 are reported in Table 3 and informed the interprofessional expert design workshop.

**Table 3:** Results from Delphi survey regarding clinically important pain inputs for the app.

Pain assessment or pain tracking item		Is this item:		
		Necessary, n (%)	Desirable, n (%)	Not needed, n (%)
	Have you had pain since your last entry?^a^	11 (79)	3 (21)	0 (0)
	How much pain do you have right now?	14 (100)	0 (0)	0 (0)
	Show your worst pain since your last entry	11 (79)	3 (21)	0 (0)
	When you had your worst pain, how long did it last?	10 (72)	3 (21)	1 (11)
	Show on body map where you hurt	12 (86)	2 (14)	0 (0)
	Does your pain bug, annoy, or bother you right now?	9 (64)	3 (21)	2 (14)
	Select the words that best describe your pain	10 (71)	4 (29)	0 (0)
**Does your pain:**				
	Make you feel mad or angry?	6 (42)	4 (29)	4 (29)
	Make you feel sad?	5 (36)	6 (43)	3 (21)
	Make you feel worried?	4 (29)	8 (57)	2 (14)
	Affect your sleep?	13 (93)	1 (7)	0 (0)
	Get in the way of you doing things?	13 (93)	1 (7)	0 (0)
	Affect being able to move around (eg, walking)?	14 (100)	0 (0)	0 (0)
	Affect your eating or drinking?^a^	11 (79)	3 (21)	0 (0)
Do you have any other symptoms related to your pain?^a^		11 (85)	2 (15)	0 (0)
Have you taken any pain medicine?		14 (100)	0 (0)	0 (0)
Did your pain medicine cause any side effects?		9 (64)	4 (29)	1 (7)
What other strategies did you use to try and reduce your pain?		10 (71)	4 (29)	0 (0)
Show how well you felt you were able to manage your pain^a^		10 (77)	2 (15)	1 (8)
Is there anything else you want to tell us about your pain?		10 (72)	2 (14)	2 (14)

^a^n=13 participants.

**Figure 2:**
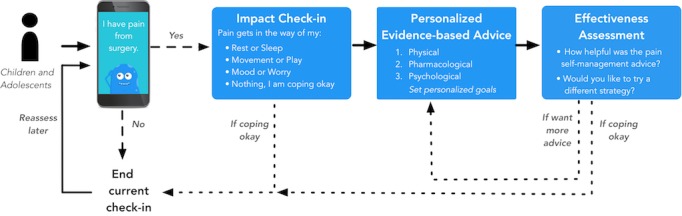
iCanCope PostOp pain care algorithm.

#### Interprofessional Expert Design Workshop

Data generated at the workshop were synthesized into a preliminary app pain care algorithm (Figure 2) that provides a framework for app system requirements for (1) clinically important postoperative pain inputs by children and adolescents and (2) evidence-based pain self-management strategies.

#### Clinically Important Pain Inputs by Children and Adolescents

Discussion about clinically important pain inputs focused on the 9 pain-assessment or pain-tracking items identified as “necessary” by more than or equal to 75% of participants in the Delphi survey (Table 3). These items focused on the presence of pain, current and worst pain intensity, pain location, and pain impact on function. A tenth item regarding use of pain medications was discussed as part of app pain management advice.

The use of validated self-report pain intensity scales was considered, such as an 11-point numeric rating scale or faces scales; however, participants identified that the primary goal of pain assessment within the app was to identify the need for automated generation of pain advice. It would be difficult to rely on scores from numeric or faces pain rating scales, given their idiosyncratic meaning to determine when pain advice should be pushed within the app. As 1 health care provider stated:

When we ask about pain with a number, it doesn’t mean anything...when we ask about pain it should be none, bearable, unbearable...[or] I have pain but it’s tolerable. This would then tie it to impact.

PP-3

Another health care provider also said:

[I] strongly suggest not using a number because that [pain] threshold is going to be different for every kid. So, it’s the impact and whether they are doing things to get better.

PP-11

Participants considered pain location and pain quality descriptors as pain inputs to inform whether pain was from the surgical site or to identify the type of pain (eg, musculoskeletal versus neuropathic). However, with input from mHealth design experts, participants deemed it would be difficult to easily and meaningfully synthesize this information in an automated way. The app needs to generate appropriate advice based on inputs by the end user. Although pain locations and descriptors may vary significantly, participants identified that pain management advice would be more effectively tailored to the functional impact of pain. As such, pain location and pain descriptors were omitted as pain inputs from the pain care algorithm.

Participants recommended self-reported assessment and tracking of the impact of pain on function and recovery after surgery, particularly in areas where useful self-management information could be generated by the app. Identified areas included impact of postoperative pain on rest or sleep, movement (including play), and emotions (such as mood and anxiety). As a person living with chronic postoperative pain stated:

I think it’s really important to find out how the patient is feeling. Whatare their emotions? I know I had surgery...when I woke up, I wasn’t expecting the pain levels that I had and I was really worried, I was really concerned.

PP-10

A health care provider added:

[It] would be important to include questions that can trigger a response.

PP-4

Finally, participants recommended reassessment of pain and impact. Participants discussed frequency of assessments and tracking within the app (range every 4 to 12 hours) with potential tapering with greater time (days) since surgery. Participants recommended a pain and impact reassessment to judge effectiveness of generated pain advice. This approach would inform pain management strategies to be pushed to the user in future or the need to suggest a different strategy if not effective. Participants also recommended that the app should generate advice to seek additional support from a health care provider or hospital should the user report repeated high levels of pain or functional difficulty over the course of several days when postoperative pain reduction and improved function would typically be expected (eg, 5 days after day surgery).

#### Evidence-Based Pain Management Advice

As endorsed by children, adolescents, parents, and health care providers in phase 1, participants at the workshop generated pain management advice for the app that could be self-directed by the child or adolescent with minimal training, previous experience, or involvement or direction of an adult, such as a parent or health care provider. A biopsychosocial approach to the management of postoperative pain was recommended and used as a framework to group pain management advice into pharmacological, psychological, and physical strategies, in addition to education [36]. A list of specific strategies was generated from available research evidence in pediatric postoperative, acute, and chronic pain [4,35] and clinical expertise. Participants recommended key strategies to be pushed based on assessed areas of pain impact (Table 4). Given the focus of the app on self-management, participants deemed it would be too difficult and inappropriate to provide individualized advice regarding frequency or dosing of pain medications in the app. Furthermore, integration of medication management into the app would have implications regarding classification of the app as a medical device, thus potentially limiting its accessibility via health care provider only instead of directly to the public via the consumer app store [37]. As such, a generic recommendation regarding medications was proposed with a greater emphasis on psychological, physical, and education strategies. Participants recommended introducing the app before surgery, such as during preoperative appointments, to prepare for postoperative pain management (eg, to identify or practice preferred strategies).

**Table 4:** Summary of expert-generated pain self-management strategies for the app.

Category, pain management advice^a^		Area of pain impact self-reported in the app		
		Rest or sleep	Movement or play	Mood or worry
**Psychological**				
	Deep breathing	✓^b^	✓	✓
	Active distraction *(eg, reading, talking to a friend, and videogames)*	—^c^	✓	✓
	Passive distraction *(eg, listen to music or watch television or a movie)*	✓	✓	—
	Imagery	✓	✓	✓
	Mindfulness	—	—	✓
	Cognitive restructuring	—	—	✓
	Coping talk	—	✓	✓
	Humor	—	—	✓
	Meditation	✓	—	✓
	Relaxation	✓	—	—
**Physical**				
	Movement	✓	✓	✓
	Comfort positioning	✓	—	✓
	Heat	✓	✓	—
	Cold	✓	—	—
	Pacing	—	✓	✓
	Walking or upright activities	—	✓	✓
	Massage	✓	✓	—
**Pharmacological**				
	Pain medication *(eg, “Take medications as prescribed. If you have any concerns, speak to your healthcare team.”)*	✓	✓	—
**Education**				
	Pain	✓	✓	✓
	Surgery	✓	✓	✓
	Other (*eg, sleep*)	✓	✓	✓

^a^Examples provided in italics.

^b^Applicable.

^c^Not applicable.

## Discussion

### Summary of Findings

This paper presents 2 early phases applying a user-centered design approach to developing an evidence-based mHealth app for self-management of pediatric acute postoperative pain. The needs assessment with children and adolescents, their parents, and health care providers identified challenges to acute postoperative pain care in the home setting that they felt could be addressed through a smartphone-based self-management app. All participants reported the 3 proposed features of the app as important (pain tracking, pain advice, and goal setting). Pain tracking can improve information and communication regarding pain and related symptoms to inform personalized intervention planning and tailoring. Not surprisingly, the needs assessment revealed a heavy reliance on medication for postoperative pain management. Pain advice within the app can increase children’s and adolescents’ independence and ability to self-manage their own symptoms after surgery as well as increases accessibility to a greater variety of evidence-based pain management interventions beyond the primary reliance on medications to include physical, psychological, and education strategies [4,36]. Finally, goal setting offered potential individual tailoring in identifying and moving toward realistic postoperative recovery. Comprehensively, these features address core disease self-management tasks of medical, behavioral, emotional, and role management [15]. Additional features of direct communication with health care providers and medication tracking were proposed. These features were considered during the subsequent interprofessional expert design workshop and attempts made to address these needs within feasible and sustainable limitations such as project budget. However, due to concerns about reduced feasibility and goal of widespread sustainable implementation of the app across clinical care settings, the potential integration of these features will require continued consideration within future iterations of the *iCanCope PostOp* app.

The Delphi survey and interprofessional expert design workshop were built on the needs assessment by tangibly formulating the app functions of pain tracking and pain advice endorsed by children, adolescents, parents, and health care providers. Specifically, the Delphi survey and design workshop led to the successful and efficient generation of a complete preliminary pain care algorithm for the *iCanCope PostOp* app, including clinically relevant inputs for feasible assessment and reassessment (tracking) of pain and function, as well as a catalog of pain management advice to be pushed to end users. This collaboratively developed algorithm integrated end users’ (children and adolescents) identified needs and perspectives with research evidence and interprofessional clinical expertise. The final list of clinically important pain inputs includes some, but not all, of the suggested elements of postoperative pain assessment, for example, omitting pain location and quality, and aggravating factors [4]. It potentially facilitates more accurate tracking of pain onset and patterns, functional impact, and identification of effective treatments. Despite the commonplace nature of acute postoperative pain in children, relatively limited research has addressed how to best manage it [4,35]. As such, in generating the list of pain advice, experts also drew on their knowledge of evidence-based strategies for managing acute and chronic nonsurgical pain. Furthermore, the need to provide individualized medication information via a self-management app presented with substantive challenges, leading to the development of generic pharmacological advice despite evidence of an identified need [13].

Our previously conducted scoping review of available postoperative pain apps identified that none of the apps were comprehensive in terms of core disease self-management features nor addressed key features of recommended postoperative pain care [20]. Furthermore, they had poor scientific foundation, lacked involvement of end users in their development, and none were designed for pediatric populations. This evidence suggests that currently available apps for postoperative pain are likely to perpetuate poor uptake and unclear effectiveness of apps for pain despite increasing interest [21-23]. Participants identified areas of need for pediatric patients that are currently poorly addressed in clinical practice guidelines for postoperative pain management, given a dearth of empirical evidence in pediatric populations [4]. Specifically, guidelines focus on education regarding medication tapering in discharging postoperative patients from hospital; however, children, adolescents, and health care providers identified challenges with pain and symptom assessment and interventions that were readily integrated into the *iCanCope PostOp* app design. The application of user-centered design ensures that *iCanCope PostOp* will address the context-specific patient-perceived problems in managing acute postoperative pain at home.

### Strengths and Limitations

The needs assessment was conducted with a convenience sample of children and adolescents who had recently undergone surgery and their parents. Although this group includes diverse types of surgeries, it is potentially limited by a lack of comprehensiveness of all types of surgeries for potential end users of the *iCanCopePostOp* app. Subsequent iterative stages to be completed in the user-centered design process (Figure 1) will allow confirmation and/or modification as needed with a more diverse group of end users. Given that a smartphone app and potential app features were proposed to participants in the needs assessment, it is also possible that their responses supporting the use of an app could reflect social desirability bias or observer-expectancy bias. However, assessment of multiple key stakeholders’ perspectives (parents and health care providers) increases the credibility and likelihood of app recommendation and use. Despite some empirical literature on pediatric postoperative pain, recent guidelines for the management of postoperative pain suggest a significant dearth in available scientific evidence in pediatric populations [4], providing rationale for beginning with user-centered design phases of concept ideation and generation. Strengths of this work are the inclusion of pediatric patients, parents, and health care providers from 2 pediatric tertiary care centers in the needs assessment as well as international interprofessional representation of experts and people living with chronic postoperative pain in developing the pain care algorithm.

*iCanCope PostOp* is being developed as part of a larger self-management platform for youth with persistent pain, called *iCanCope*. *iCanCope* has been iteratively developed as per a user-centered design approach initially for youth with chronic pain [29], with subsequently developed iterations for arthritis and sickle cell disease currently undergoing evaluation. Sustainability is a key benefit of developing *iCanCope PostOp* as part of the larger *iCanCope* platform. *iCanCope* is designed to leverage economies of scale such that any new feature expansions benefit the whole platform. This integration enables *iCanCope PostOp* to benefit from updates and revisions completed as part of the development of other iterations of the *iCanCope* platform as relevant and applicable. Thus, updates and revisions to *iCanCope* require fewer resources (time and cost) to complete and are applied across the platform to all iterations as appropriate. Similarly, *iCanCope PostOp* benefits from the existing back-end infrastructure, design, and functionalities developed for previous iterations of the *iCanCope* platform and can build on top of it.

### Conclusions

This work completes the crucial first stage of concept generation and ideation in the user-centered design process fundamental to the subsequent design, development (iOS or Android), evaluation, and implementation of the *iCanCopePostOp* app. Future phases include vetting of the expert-generated pain care algorithm from the perspective of app end users (children and adolescents), parents, and health care providers; usability testing and design sessions with end users to develop and refine the app prototype and final product; evaluation of app effectiveness for postoperative pain care; and app implementation and public deployment [24,25]. A similar user-centered design approach can be effectively applied in the development and design of mHealth apps to address self-management needs for other pediatric conditions.

## Abbreviations

CIHR: Canadian Institutes of Health Research
mHealth: mobile health
